# Antidepressant Effect of an Orally Administered Dipeptide Mimetic of the Brain-Derived Neurotrophic Factor

**Published:** 2018

**Authors:** P. Y. Povarnina, T. L. Garibova, T. A. Gudasheva, S. B. Seredenin

**Affiliations:** Federal State Budgetary Institution “Research Zakusov institute of pharmacology”, Baltic Str., 8, Moscow, 125315, Russia

**Keywords:** dimeric dipeptide BDNF mimetic, GSB-106, depression, oral dosage form

## Abstract

Involvement of BDNF in the regulation of neuroplasticity and neurogenesis in the hippocampus, impairment of which underlies the pathophysiology of depression, makes this endogenous protein a promising object for the development of new-generation antidepressants with a neurophysiologically based mechanism of action. A low-molecular-weight BDNF mimetic, GSB-106 (a substituted dimeric dipeptide, bis-(N-monosuccinyl- L-seryl-L-lysine) hexamethylenediamide), was designed and synthesized at the Zakusov Institute of Pharmacology. GSB-106 was found to activate BDNF-specific TrkB receptors and their main post-receptor signaling pathways MAPK/ERK and PI3K/AKT. GSB-106 exhibited pronounced antidepressant activity in a rodent test battery at a dose of 0.1 to 1.0 mg/kg administered intraperitoneally. Because oral administration is preferable in the treatment of depression, which is associated with a prolonged duration and outpatient character of pharmacotherapy, we examined the antidepressant properties of GSB-106 administered orally as a pharmaceutical substance (PS) and in tablet dosage form (TDF). In the study, we used the Porsolt forced swim test in rats; a conventional antidepressant, Amitriptyline, was used as a reference drug. The antidepressant activity of GSB-106 was found to retain upon oral administration and to manifest at doses of 0.5–5.0 mg/kg for PS and 0.01–5.0 mg/kg for TDF. The effective dose of TDF was 50-fold lower than that of PS, and the efficacy of tableted GSB-106 exceeded that of Amitriptyline, the “gold standard” in antidepression care. Therefore, GSB-106, both as a substance and as a tablet dosage form, exhibits antidepressant activity when administered orally, which makes it a promising antidepressant agent, the first in the class of BDNF mimetics.

## INTRODUCTION

Experimental and clinical data indicate a central role for the brain-derived neurotrophic factor (BDNF) in the pathogenesis of depression [1]. The antidepressant activity was observed in experiments with intracerebral administration of BDNF [2-4]. The BDNF level is reduced in the blood plasma of patients with depression; after treatment with antidepressants, the BDNF level returns to normal values [5]. In addition, a decreased BDNF level was detected in the hippocampus of patients with depression and in suicide victims [6, 7]. The pathogenetic role of BDNF in depression has been demonstrated to be associated with the regulation of neuroplasticity and neurogenesis in the hippocampus [8], impairment of which is considered to be the leading etiopathogenetic factor of the disease [9].

Obviously, BDNF is an important object in the development of antidepressants with a pathophysiology-based mechanism of action.

The physiological effects of BDNF, including the regulation of neurogenesis and maintenance of neuroplasticity, are mediated by TrkB receptors, with MAPK/ERK and PI3K/AKT being the main pathways of their signal transduction [10, 11]. We have developed low-molecular-weight compounds that mimic the beta-turns of some BDNF loops and experimentally proved that they selectively activate post-receptor cascades [12, 13]. In this case, BDNF mimetics selectively activating either PI3K/AKT or MAPK/ERK were found not to exhibit antidepressant activity, which indicates that both post-receptor signaling pathways are essential for this activity.

It should be noted that the clinical use of native BDNF is limited due to its unsatisfactory pharmacokinetic properties.

In this regard, the dipeptide GSB-106, a mimetic of the BDNF loop 4 beta-turn (*bis*-(N-monosuccinyl- *L*-seryl-*L*-lysine) hexamethylenediamide), was selected as a promising antidepressant at the Zakusov Institute of Pharmacology. Western blot analysis revealed that GSB-106 activates BDNF-specific TrkB receptors and their post-receptor signaling pathways MAPK/ERK and PI3K/AKT [12, 16]. GSB-106 exhibited pronounced antidepressant activity in a rodent test battery at a dose of 0.1 to 1.0 mg/kg administered intraperitoneally [15, 17]. Probably, the mechanisms of GSB-106 antidepressant action, like those of the full-length protein, are associated with neurogenesis and synaptogenesis. The effect of GSB-106 on neurogenesis was previously demonstrated in a mouse predator stress model [18].

Obviously, the oral dosage form is preferable in depression, which is associated with a prolonged duration and outpatient character of pharmacotherapy. Therefore, we studied the antidepressant properties of GSB- 106 administered orally both as a pharmaceutical substance (PS) and in tablet dosage form (TDF).

## EXPERIMENTAL

**Medicinal products**

GSB-106 PS was synthesized at the Department of Pharmaceutical Chemistry of the Zakusov Institute of Pharmacology as described earlier [15]. GSB-106 TDF for oral use was developed and produced at the Experimental and Technological Department of the Zakusov Institute of Pharmacology and contained 1% of GSB-106 PS and 99% of a filler consisting of lactose, microcrystalline cellulose, polyethylene glycol-polyvinyl alcohol copolymer, and magnesium stearate.

Amitriptyline in an injectable dosage form was produced at the Moscow Endocrine Plant (Russia).

**Animals**

Experiments were performed on 188 white outbred male rats weighing 240–270 g received from the Stolbovaya Branch of the Scientific Center of Biomedical Technologies of the Federal Medical Biological Agency. The animals were kept at the vivarium with a natural light cycle and free access to standard pellet feed and water in accordance with Sanitary Regulations 2.2.1.3218-14 Sanitary and epidemiological requirements for organization, equipment, and maintenance of experimental biological clinics (vivaria) of August 29, 2014, No. 51. The study was organized and implemented in accordance with Order of the Ministry of Health of Russia No. 199 of April 1, 2016 On Approval of the Rules for Good Laboratory Practice. The experiments were approved by the Biomedical Ethics Commission of the Zakusov Institute of Pharmacology (Protocol No. 2 of February 20, 2017).

**Investigation of antidepressant activity**

The antidepressant activity of GSB-106 in rats was determined in the Porsolt forced swim test [19, 20]. The setup for assessing antidepressant activity consisted of six cylindrical vessels, each 20 cm in diameter and 60 cm in height, separated by opaque walls. The vessels were filled with 40 cm of water at a temperature of 22°C. First, each animal was placed in a vessel with water for 15 min, after which the animal was left to dry and then returned to its home cage. After 24 h, the animals were placed in water for 5 min to assess the time of a characteristic immobile posture (no active struggle behavior). The behavior of the animals was recorded with a video camera. Video recordings of the experiments were processed in semi-automatic mode using the RealTimer software (Open Science, Russia).

**Experimental design**

GSB-106 PS was dissolved in distilled water and administered to rats daily for 14 days at doses of 0.1, 0.5, 1, 5, and 10 mg/kg orally in a volume of 1 mL/kg. Control animals received distilled water in the same regimen. GSB-106 TDF was suspended in a 1% starch solution and administered to rats daily for 14 days at doses of 0.001, 0.01, 0.05, 0.1, and 5.0 mg/kg orally in a volume of 1 mL/kg. The control animals received a placebo in the same regimen, which was a suspension of the TDF filler in a 1% starch solution. The classical tricyclic antidepressant Amitriptyline was used as a reference drug; it was diluted in distilled water and administered orally to rats at a dose of 5.0 mg/kg [21] in a volume of 1.0 mL/kg for 14 days. The control animals were administered distilled water in the same regimen. Twenty-four hours after the last administration of the preparations, the rats were put into cylinders with water to foster a depressive-like state; after another 24 hours, testing was performed.

**Statistical analysis**

Intergroup differences were assessed using the Student’s *t*-test and the Mann-Whitney U test (the Bonferroni adjustment was used if more than two groups were compared). Differences were considered statistically significant at *p *≤ 0.05. The data were presented as means and standard errors of the means.

## RESULTS

**Antidepressant activity of orally administered GSB-106 PS**

In the Porslot forced swim test, the control rats receiving distilled water, after a period of activity, adopted a characteristic immobility posture (no active struggle behavior) and retained it with small interruptions until the end of the experiment. The immobility time in the control animals in different experiments ranged from 178 to 216 s, on average. GSB-106 PS at doses of 0.5, 1.0, and 5.0 mg/kg statistically significantly (*p* ≤ 0.05) reduced the immobility time compared to that in the controls (*Table*), 1.5-, 1.2-, and 1.6-fold, respectively, which was an indication of the presence of antidepressant activity. At doses of 0.1 and 10 mg/kg, GSB-106 had no antidepressant activity, which was an indication of the effect’s dependence on the dose. In this case, the effect of GSB-106 PS at a dose of 0.5 mg/kg was comparable to that of Amitriptyline at a dose of 5.0 mg/kg (per os) (*Table*).

**Table:** Antidepressant-like effects of orally administered GSB-106 in a Porsolt forced swim test in rats

Group	n	Dose, mg/kg	Immobility, s	Decrease in the immobility time, as a fraction of control
Antidepressant effect of GSB-106 PS				
Control (water)	8		177.9 ± 9.3	
Amitriptyline	8	5.0	134.5 ± 10.1*#	0.77
GSB-106	8	0.1	184.8 ± 16.6	
	8	0.5	116.9 ± 21.6#	0.67
Control (water)	18		199.2 ± 10.0	
GSB-106	18	1.0	163.2 ± 7.9#	0.83
Control (water)	10		216.3 ±13.8	
GSB-106	10	5.0	190.8 ± 9.8	0.63
Control (water)	10		190.8 ± 9.8	
GSB-106	10	10.0	202.2 ± 14.9	
Antidepressant effect of GSB-106 TDF				
Control (placebo)	10		245.7 ± 13.4	
GSB-106	10	0.001	192.5 ± 13.2	
GSB-106	10	0.01	155.2 ± 20.3**	0.63
GSB-106	10	0.05	134.9 ± 18.8***	0.56
GSB-106	10	0.1	165.4 ± 15.1**	0.67
Control (placebo)	10		201.2 ± 12.1	
GSB-106	10	1.0	159.9 ± 23.6#	0.77
GSB-106	10	5.0	92.8 ± 17.2***	0.45

Note. n is the number of animals in a group. Data are presented as means and standard errors of the means.

^*^ – p ≤ 0.05;

^**^ – p < 0.01;

^***^ – p < 0.001 compared to the control group (Mann-Whitney U test);

^#^ – p ≤ 0.05 compared to the control group (Student’s t test).

Therefore, GSB-106 PS was found to trigger antidepressant activity upon oral administration at a dose of 0.5–5 mg/kg.

**Antidepressant activity of orally administered GSB-106 TDF**

The immobility time in the control rats receiving placebo was 246 and 201 s in two experiments. In the Porsolt forced swim test, orally administered GSB-106 TDF at a dose of 0.01, 0.05, 0.1, and 5.0 mg/kg statistically significantly (*p * < 0.01) reduced the immobility time in rats compared to that in the controls 1.6-, 1.8-, 1.5-, and 2.2-fold, respectively (*Table*). The effect of GSB-106 TDF was dose-dependent and was absent at a dose of 0.001 mg/kg.

## DISCUSSION

The study revealed that the antidepressant activity of dipeptide BDNF mimetic GSB-106, which had been earlier detected upon intraperitoneal administration at doses of 0.1–1.0 mg/kg, was retained upon oral administration and was exhibited at doses of 0.5–5.0 mg/kg for PS and 0.01–5.0 mg/kg for TDF of GSB-106. GSB-106 TDF was active at 50-fold lower doses than GSB-106 PS, and the efficacy of GSB-106 TDF exceeded that of Amitriptyline, the gold standard in antidepression care.

It is important that the activity of orally administered GSB-106 is comparable to that of intraperitoneal administration. This stability of GSB-106 in biological media is associated with the absence of bond targets for proteases, aminopeptidases, and carboxypeptidases (due to its dipeptide nature) and protected N- and C-termini, respectively. This confirms the prospects and advantages of substituted dipeptides as oral medication compared to oligopeptides that easily degrade in the gastrointestinal tract and do not penetrate the gastrointestinal barrier. Therapeutic oligopeptides affecting the central nervous system are used mainly in intranasal forms, and oligopeptides exhibiting peripheral effects are used in injectable forms. However, dipeptide drugs, such as the nootropic agent Noopept (N-phenylacetyl-*L*-prolylglycine ethyl ester), the antipsychotic drug Dilept (N-caproyl-*L*-prolyl-*L*-tyrosine methyl ester), and the anxiolytic agent GB-115 (N-phenylhexanoyl-glycyl-*L*-tryptophan amide) have been demonstrated to retain their activity upon oral administration [22, 23]. As found earlier [24, 25], there are specific transport systems for the transfer of dipeptides through the enteral mucosa of the gastrointestinal tract (PEPT-1) and through the blood-brain barrier (PEPT-2).

Therefore, the GSB-106 dipeptide and its tablet dosage form possess antidepressant activity, which makes GSB-106 an original antidepressant drug, the first in its class.

## CONCLUSION

The dimeric dipeptide BDNF mimetic GSB-106 exhibits antidepressant activity when administered orally. The developed dosage form of GSB-106 is superior to the pharmaceutical substance both in doses and in antidepressant effect.

The study results demonstrate the reasonability of developing a dipeptide GSB-106-based drug that, based on its dipeptide structure and BDNF-ergic mechanism of action, may be classified as first in its class.

## Abbreviations

BDNF: brain-derived neurotrophic factor
PS: pharmaceutical substance
DF: dosage form
